# Reproductive disorders in dairy cattle under semi-intensive system of rearing in North-Eastern India

**DOI:** 10.14202/vetworld.2016.512-518

**Published:** 2016-05-26

**Authors:** M. H. Khan, K. Manoj, S. Pramod

**Affiliations:** 1 ICAR-National Research Centre on Mithun, Jharnapani, Medziphema - 797 106, Nagaland, India; 2 ICAR Research Complex for NEH Region, Umiam - 793 103, Meghalaya, India; 3 Central Institute for Research on Cattle, Meerut - 255 001, Uttar Pradesh, India

**Keywords:** anestrus, dairy, infertility, Meghalaya, reproductive disorders

## Abstract

**Aim:**

This study was conducted to determine the incidence of major reproductive problems of dairy cattle reared under a semi-intensive system by small and marginal farmers in Meghalaya province of North-Eastern India.

**Materials and Methods:**

In a 3 years study, a total of 576 crossbred dairy cattle (212 Holstein Friesian cross and 364 Jersey cross) from all districts (n=11) of Meghalaya were assessed with the survey, clinical examination, and personal observations.

**Results:**

Out of the total animal assessed, 33.85% (n=195) were found to be affected with one or more of the clinical reproductive problems. Repeat breeding (RB), anestrus, retention of fetal membrane, and abortion were found to be the major clinical reproductive problems. Out of the total animal affected with reproductive disorders, the incidence of anestrus, RB, retention of fetal membrane, and abortion was found to be 31.79% (n=62), 24.61% (n=48), 14.35% (n=28), and 11.25% (n=22), respectively. In addition, dystocia (5.12%), prolapse (1.53%), endometritis (4.61%), and pyometra (6.66%) were minor clinical reproductive problems. There was a significant difference in the incidence of reproductive disorders with respect to breed, age, and parity.

**Conclusion:**

It was revealed from this study that RB, anestrus, retention of fetal membrane, and dystocia are the major clinical reproductive problems in Meghalaya. Results indicated unsatisfactory feeding, housing, and health management practices are the main cause of low fertility of dairy cows. Lack of scientific knowledge, low access to breeding, and health services further contributed to low productivity and fertility.

## Introduction

Meghalaya, the North-Eastern province of India, is located between 20°1′ and 26°5′ North latitudes and 85°49′ and 92°52′ East latitudes. The altitude varies from 30 to 2000 m MSL. The state has rich natural resources, and the climate ranges from tropical and subtropical to temperate. The state has almost 69.7% forest coverage and receives heavier rainfall (average rainfall 2000 mm) [1]. The annual maximum temperature ranges from 10°C to 20°C during winter and 25-35°C during summer season over different places [1].

The animal husbandry is the main component of agriculture development in India and particularly in North-Eastern part of the country owing to the hilly terrain and undulating topography where agriculture is largely rain fed. Dairy production in this part of the country has not been viewed seriously because of less intake of milk and milk products by the native tribal people [2]. Most of the dairy farming is being done by the outside settlers mainly from neighboring states or Nepal. In Meghalaya, the crossbred cattle population is only 26458 in number which is 0.67% of India’s crossbred cattle population [3]. Over the last 5 years showed 3.94% increase in crossbred cattle population which is lower than the national average (7.58%) [3]. The farmers generally practice mixed crop-livestock production system with crop mostly being the primary production, and the animals are generally reared under semi-intensive system [4]. The smallholder’s dairy sector in this part of the country is facing with several challenges. These include limited breeding stock, high cost of feed, non-availability of green fodder round the year, poor management, inadequate health extension services which ultimately resulted in low productivity, and poor fertility or infertility of dairy cows. Reproductive disorder among farm animals is the great economic problem. It is particularly widespread among dairy cattle [5]. Economy of the dairy farming largely depends on pregnancy rate after insemination. The 12-month calving interval is advantageous for high milk yield per cow with the good economic return. It is accepted that bovine genital infections, either specific or non-specific accounts for a large number of pregnancy failure in cows [6]. These reproductive health problems are the bottleneck in the production process and productivity in the livestock sector.

Therefore, it is justifiable to generate scientific information and database on the production system, and the major reproductive problems of dairy cows in the study area with the objective to determine the prevalence of major infertility problems of dairy cattle. It is anticipated that the information generated could be used as a basis for interventions to improve dairy cattle productivity among smallholder farmers in Meghalaya.

## Materials and Methods

### Ethical approval

The present study was conducted in accordance with the guidelines set by Animals Ethics Committee of the Institute.

### Study area and animals

This study was conducted at Indian Council of Agricultural Research Complex for North-Eastern Hill Region, Barapani, Meghalaya, India. The study was conducted for 3 years from 2010 to 2013 to determine the prevalence of reproductive diseases in randomly selected 576 crossbred dairy cattle covering all districts of Meghalaya, India. All the animals selected for the study were reared in semi-intensive type of management where animals were stall-fed with little open area for grazing. Almost same cattle rearing pattern in terms of feeding, housing, and health care management was seen across the study area. Feed comprised broken rice, wheat/rice bran, maize, and vegetables mixed and boiled. Feed was provided twice daily. Apart from this, paddy straw and some green fodder grass were also given.

### Data collection on reproductive disorders

Crossbred dairy cows (n=576) were subjected to gynecological examinations to find out the prevalence of reproductive disorders. The detailed history of the cow, including the date of previous artificial insemination (AI) or natural mating, was obtained from the cattle owner through structured questionnaire format. Diagnosis of reproductive diseases was made on the basis of history, clinical signs, and response of the treatment. The cows having apparently normal genitalia but failed to conceive through natural mating with fertile bull or using AI with quality semen consecutively for three times were considered as repeat breeders. The cows with the history of prolonged absence of estrus were examined twice at 11 days apart and those showing the smooth ovaries on both the examinations were confirmed as anestrus [7].

### Biochemical analysis of blood serum

Out of 576 cows, blood sample of 240 dairy cattle was collected for estimation of total protein, albumin, and cholesterol concentration in blood serum. The animals were categorized into three group, *viz*., normal breeding (without any apparent reproductive disorder), repeat breeding (RB), and anestrus comprising of 80 animals in each group. 10 ml of blood samples were collected from jugular vein without adding anticoagulant. Serum was separated by centrifugation and transferred into a sterilized plastic vial and labeled. The serum samples were used immediately for glucose estimation using standard kit. The samples, which were not able to be analyzed on the same day of collection, were stored at −20°C until analyzed. The total protein, albumin and cholesterol concentration were estimated using the standard kit (Qualigens Diagnostic, Glaxo, Mumbai, Maharashtra, India) following the procedure given by the manufacturer.

### Data processing and analysis

The collected data were sorted manually, and questionnaires were coded before processing. The data were also checked thoroughly for consistency and to make sure that there was no missing value. Animals were classified into two genotypes, *viz*., Holstein Friesian (HF) crossbred (HF×Indian local cattle; n=212) and Jersey crossbred (Jersey×Indian local cattle; n=364). The data were further categorized into the basis of age group and parity. There were four age groups, *viz*., <3 years (n=132), 3-5 years (n=170), 5-7 years (n=189), and >7 years (n=85). Similarly parity wise, four groups, *viz*., heifers (n=147), 1^st^-3^rd^ parity (n=234), 4-6^th^ parity (n=145), and >6^th^ parity (50) were classified. The prevalence and relative frequencies of reproductive health problems were determined as the proportion of affected animals out of the total animals examined and the total number of particular disorder out of the total affected animals, respectively. The baseline survey data were entered and analyzed in SPSS 15.0 program. Descriptive statistics was done to explore the prevalence of reproductive diseases and Duncan *t*-test was used to determine the level of significance among clinical cases of cow. Blood biochemical profile data were subjected to one-way analysis of variance.

## Results

### Incidence of reproductive disorders

All the animals selected for the study were maintained under the semi-intensive system of management. As the animals were provided shelter, concentrate feed and water by the owner but allowed to graze in open for green fodder. The result showed that out of total 576 dairy cows examined, 33.85% (n=195) were found to be affected either with one or more reproductive health problems. Reproductive disorders reported in this study were anestrus (31.79%), RB (24.61%), retention of fetal membrane (14.35%), abortions (11.25%), dystocia (5.12%), prolapse (1.53%), endometritis (4.61%), and pyometra (6.66 %) irrespective of breed, age, and parity of the cow (Table-1). Anestrus syndrome was the most common cause of infertility of cow. In this study, RB was found to be the second highest common reproductive disease which was 26.66%. The major reproductive problems were anestrus, RB, retention of fetal membrane, and abortions which contributed >80% of the total affected animals. However, dystocia, vaginal prolapse, endometritis, and pyometra also found to affect the fertility of dairy cattle.

**Table-1:** The relative frequency of various reproductive disorders in Meghalaya.

Reproductive disorders (n=195)	Frequency	Percent of total affected animals
Anestrus	62	31.79
RB	48	24.61
RFM	28	14.35
Abortion	22	11.25
Dystocia	10	5.12
Prolapse	03	1.53
Endometritis	09	4.61
Pyometra	13	6.66
Total	195	100

RFM=Retained fetal membrane, RB=Repeat breeding

### Effect of breed on reproductive disorders

Out of total 576 animals examined in this study, HF cross showed a higher incidence of reproductive disorders (43.39% versus 28.29%) than Jersey crosses (Table-2). Breed had a significant effect on anestrus, abortion, dystocia, and endometritis. No significant difference was observed in cases of RB, retained fetal membrane (RFM), prolapse, and pyometra. The incidence of anestrus, abortions, dystocia, and endometritis was significantly higher in HF crossbred cattle than in Jersey cross which was found to be 11.32% versus 6.59%, 5.66% versus 2.74%, 6.13% versus 1.92%, and 2.83% versus 0.82%, respectively (Table-3).

**Table-2:** Reproductive disorders in HF and Jersey crossbred cows.

Breed	Total number of cows examined	Number of cows affected	Number of non-affected cows	Percent affected (%)
HF cross	212	92	120	43.39
Jersey cross	364	103	261	28.29
Total	576	195	381	33.85

HF=Holstein Friesian

**Table-3:** Effect of breed on the prevalence of reproductive diseases of dairy cows.

Reproductive disorders	Breed N (%)		Total (n=576) N (%)	Level of significance

	HF cross (n=212)	Jersey cross (n=364)		
Anestrus	24 (11.32)	24 (6.59)	48 (8.33)	*
RB	25 (11.79)	37 (10.16)	62 (10.76)	NS
RFM	14 (6.60)	14 (3.84)	28 (4.86)	NS
Abortion	12 (5.66)	10 (2.74)	22 (3.81)	*
Dystocia	13 (6.13)	07 (1.92)	10 (1.73)	*
Prolapse	02 (0.09)	01 (0.27)	03 (0.52)	NS
Endometritis	06 (2.83)	03 (0.82)	09 (1.56)	*
Pyometra	07 (3.30)	07 (1.92)	13 (2.25)	NS
Total	92 (43.39)	103 (28.29)	195 (33.85)	

* Significant (p<0.05)

n=Number of observations, N=Number of animals affected, NS=Non significant (p<0.05). RFM=Retained fetal membrane, RB=Repeat breeding

### Effect of age

Age group had significant (p<0.05) effect on RB and anestrus. Lower incidence of reproductive disorders was reported in <3 years age group and no cases of prolapse, endometritis, and pyometra were recorded during the study period. On the contrary, significantly higher anoestrus cases were recorded in 5-7 and >7 years age group followed by 3-5 years age group and in heifers. RB was highest in >7 years age group and minimum in <3 years age group. No significant difference was recorded in RFM, abortion and dystocia, proplapse, endometritis, and pyometra between the age groups (Table-4).

**Table-4:** Effect of age group on the prevalence of reproductive diseases of dairy cows.

Reproductive disorders	Age group N (%)				Overall (n=576) N (%)	Level of significance

	<3 years (n=132)	3-5 years (n=170)	5-7 years (n=189)	>7 years (n=85)		
Anestrus	05 (3.48)	13 (7.64)	25 (13.22)	5 (5.88)	48 (8.33)	*
RB	05 (3.78)	20 (11.36)	22 (11.64)	15 (17.64)	62 (10.76)	*
RFM	06 (4.54)	10 (5.88)	07 (3.70)	05 (5.88)	28 (4.86)	NS
Abortion	03 (2.27)	07 (4.11)	06 (3.17)	06 (7.05)	22 (3.81)	NS
Dystocia	06 (4.54)	02 (1.17)	02 (1.05)	-	10 (1.73)	NS
Prolapse	-	-	02 (1.05)	01 (1.17)	03 (0.52)	NS
Endometritis	-	03 (1.76)	04 (2.11)	02 (2.35)	09 (1.56)	NS
Pyometra	-	05 (2.94)	04 (2.11)	04 (4.70)	13 (2.25)	NS
Total	25 (18.93)	60 (35.29)	72 (38.09)	38 (44.70)	195 (33.85)	

* Significant (p<0.05),

n=Number of observations, N=Number of animals affected, NS=Non significant (p<0.05), RFM=Retained fetal membrane, RB=Repeat breeding

### Effect of parity

Parity had a significant effect on the occurrence of various reproductive disorders under smallholder’s dairy cattle production system in Meghalaya. Maximum incidence of reproductive disorders was reported between 1^st^ and 3^rd^ parity followed by 4-5^th^ parity. Cases of RB, anestrus, RFM, and abortion were highest in 1^st^ to 3^rd^ parity followed by 4-5^th^ parity group, and minimum was reported in heifers. All cases of prolapse were reported only in 1^st^-3^rd^ parity. Similarly, cases of endometritis were not reported in heifers and animals >6 parity. The prevalence of abortion was highest (5.12) in 2^nd^ calving and the lowest (1.36%) in heifer, 1^st^ calving, 6^th^ calving, and >7^th^ calving. The prevalence of stillbirth was highest (0.7%) in 1^st^ calving and no stillbirth in heifers, 5^th^ calving, 6^th^ calving, and >7^th^ calving. Similarly, retained placenta (4.5%), metritis (1.1%), pyometra (1.1%), uterine prolapsed (0.5%), and RB (5.2%) were higher in 2^nd^ parity. Vaginal prolapsed (0.7%) and mastitis (2.8%) were highest in 3^rd^ parity (Table-5).

**Table-5:** Effect of parity on the incidence of reproductive diseases of dairy cows.

Reproductive disorders	Parity N (%)				Overall (n=576) N (%)	Level of Significance

	Heifer (n=147)	1^st^ to 3^rd^ (n=234)	4-5^th^ (n=145)	>6^th^ (n=50)		
Anestrus	5 (3.4)	26 (11.11)	14 (9.65)	3 (6.0)	48 (8.33)	*
Repeat Breeding	5 (3.4)	46 (19.65)	9 (6.20)	2 (4.0)	62 (10.76)	*
RFM	4 (2.72)	14 (5.98)	6 (4.13)	4 (8.0)	28 (4.86)	*
Abortion	2 (1.36)	12 (5.12)	6 (4.13)	2 (4.0)	22 (3.81)	NS
Dystocia	5 (3.4)	3 (1.28)	2 (1.37)	-	10 (1.73)	NS
Prolapse	-	3 (1.28)	-	-	3 (0.52)	NS
Endometritis	-	6 (2.56)	3 (2.06)	-	9 (1.56)	NS
Pyometra	2 (1.36)	7 (2.99)	3 (2.06)	1 (2.0)	13 (2.25)	NS
Total	23 (15.64)	117 (50.0)	43 (29.65)	12 (24.0)	195 (33.85)	

* Significant (p<0.05),

n=Number of observations, N=Number of animals affected, NS=Non significant (p<0.05), RFM=Retained fetal membrane.

### Blood biochemical profile

**Figure-1:**
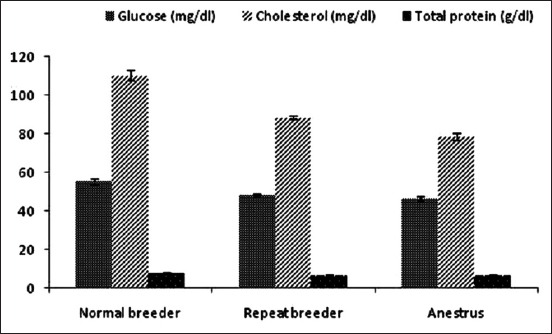
Blood biochemical profile of normal breeding, repeat breeding, and anestrus cows.

The blood serum glucose level in normal breeding, RB, and anestrus animals were 55.45*±*1.34, 48.36*±*0.75, and 46.54*±*0.99 mg/dl, respectively. The serum total cholesterol level of normal breeding, RB, and anestrus cows were 110.54*±*2.54, 88.65*±*0.96, and 78.64*±*1.79 mg/dl, respectively. Total serum protein level of normal breeding, RB, and anestrus cows were 7.54*±*0.68, 6.43*±*0.62, and 6.58*±*0.51 g/dl, respectively (Figure-1).

## Discussion

Although livestock is an important source of income for smallholders and the landless in North-East India, still it is largely deficit in egg, pork, milk, and mutton production. Products such as milk and eggs are steady source of cash income, and live animals are important natural assets for the poor, which can be easily liquidated for cash during emergency. Smallholders and landless together control 75% of the country’s livestock resources and are capable of producing at a lower cost because of availability of sufficient labor with them. Evidence shows that smallholders obtain nearly half of their income from livestock [8].

This study documented the common reproductive disorders encountered under smallholder’s dairy production system in North-Eastern part of India. Livestock in India and particularly in the North-Eastern region are raised as a part of mixed farming systems which is considered environmentally most benign and sustainable because of complementarities between crop and livestock production. Animals derive most of their feed-fodder requirement from agricultural residues and by-products, and in turn provide draught power and dung manure for cropping activities.

This study revealed 37.85% of reproductive incidence out of total animal examined which is in close approximation with earlier findings of Hadush *et al*. [9], who reported 44.3% of the cows with major pre-partum and post-partum reproductive problems in Ethiopia. Similarly in other findings of Tesfaye and Shamble [10], it was reported that out of total 231 dairy cows included in the study, 40.25% were found to be affected at least by one reproductive health problems. Anestrus (31.79%) and RB (24.61%) were two major reproductive problems encountered in more than 50% of the cows during their lifetime which are slightly higher than reported earlier [9], where the incidence of anestrus and RB as 12.9% and 11.4%, respectively. Similarly, very high incidence of anestrus (20.4%) and RB (12.8%) reported common reproductive diseases in crossbred dairy cattle in Bangladesh [11]. On the contrary, 65% of anestrus and 35% RB cases in crossbred cattle were reported in Kerala [12]. Similarly, higher prevalence of anestrus and RB (49.9% and 15.1%, respectively) in cows was reported by Serur *et al*. [13]. Major reason for anestrus in dairy cattle is due to poor quality ration provided by the farmers, lack of round the year green fodder availability, higher incidence of parasitic diseases, lack of scientific knowledge, and poor managemental practices. Higher incidence of the reproductive problem in HF cross than the Jersey cross is due to less adoptability of HF cross cattle under subtropical hill ecosystem, high milk production, and low plane of nutrition. The common cause of RB was a cystic ovarian disorder, anovulation, delayed ovulation, early embryonic mortality, improper timing of AI, lack of knowledge of proper heat detection, using old and infected bull, lack of veterinary extension services, poor quality semen, and inexperienced inseminator. Error in estrus detection and improper timings of AI was also reported by earlier workers as the common cause of RB in cows [14]. Metritis (clinical and sub-clinical), milk fever, dystocia, and retained placenta had a significant association with RB [15].

Our results are in agreement with previous findings of Tesfaye and Shamble [10], who reported the major productive health problems in the dairy cows as clinical mastitis (19.3%), abortion (9.05%), dystocia (7.75%), retained placenta (7.32%), and RB (3.87%). This variation in prevalence might be due to the differences in management and breed of the animals as well as environmental factors. The higher incidence of reproductive problems was in HF cross than in Jersey cross cattle which may be due to the fact that HF cross is less adapted to tropical conditions of high temperature and humidity, disease and low feed quality than Jersey crossbred making them more susceptible. Beside this, HF cross requires more elaborated management, feeding, and better health care than the indigenous zebu to get better reproductive performance.

Genotype had a significant effect (p<0.05) on abortion, RB, dystocia, retention of placenta, endometritis, anestrus, and milk fever. In this study, two different genetic groups, *viz*., HF and Jersey crossbred cattle were selected, and significantly higher incidence of reproductive disorders was recorded in HF cross than in Jersey. The major reason for this is the climatic conditions of Meghalaya. The climate of Meghalaya varies with the altitude. Temperature in the study area ranges between 20 and 32°C. Due to high humidity and increased temperature, HF cross is more susceptible to heat stress which may result in high incidence of reproductive disorders. Further, this climate is more conducive for the propagation of external as well as internal parasites which can easily infect the cattle through grazing. Since deworming practice is usually not being followed by the majority of farmers, the chronic parasitic infection causes loss of production and anestrus.

All the reproductive diseases were lowest in <3 years age group cows. Since the age of first calving is more than 3 years in both genetic groups. Therefore, very less incidence of reproductive disorders was found in <3 years age group. Higher incidence of reproductive diseases in 5-7 years and >7 years age group high milk production and negative energy balance. Higher incidence of RB cases in >8 years age group might be due to endocrine disturbances. Higher incidence of abortion, metritis, cystic ovaries, vaginal prolapse, uterine prolapse, RFM, dystocia, anestrus, and RB in >8 years old cows and lower in <4 and 4-6 years old cows were also reported in Bangladesh [16]. However, no significant difference was recorded in retention of placenta, abortion, dystocia, prolapse, endometritis, and pyometra between the age groups. Other factors which significantly contribute are poor body condition score, body weight, mangemental factors, environmental factors and inadequate veterinary and extension services, etc. On the contrary, it was reported that the occurrence of overall reproductive problems was not significantly affected within the different age groups. However, the problems were seen to rise with age [10].

Parity of female is one of the most important factors associated with reproductive disorders. The parity 1^st^-3^rd^ is the most productive phase of female cattle followed by parity 4^th^-5^th^. This parity falls in the age group between 3 and 8 years. Milk production is maximum up to 3^rd^ parity after that it starts declining. Due to high milk production, animals becomes in negative energy balance. As the poor farmers could not afford the high cost of concentrate feed, the productive animals fail either to return to cyclicity resulting prolonged calving to conception interval leading to anestrus or it may result into endocrine disturbances leading failure of ovulation, failure of fertilization, early embryonic mortality resulting in RB. The usual practice in this part of the country is to cull the animal after 6^th^ parity due to less milk production. The culled animals are being slaughtered and used as beef. Therefore, very less incidence of reproductive disorders after 6^th^ parity was reported. It is reported that the prevalence of RB was although found to vary from 1^st^ to 5^th^ parity, the difference was non-significant. However, significantly lowest prevalence was recorded at 6^th^ parity (6.25%) when compared to other parities [17].

It was also reported that the number of parity has significant (p<0.05) influence for the occurrence of reproductive problems where animals with higher parities were seen to show reproductive problems more frequently than those with low number of parities [10]. This study showed most of the reproductive disorders in 2^nd^ parity which is similar to the findings of earlier workers [18].

The blood serum glucose level in this study agrees with the previous findings [19]. There was a significant decrease in blood glucose level of RB and anestrus cows compared to normal breeding cows. Lower blood glucose level in RB and anestrus animals might influence the pituitary function thereby interfering the fertility [20]. The serum total cholesterol level (mg/dl) of normal breeding animals were significantly higher (p<0.05) than in RB and anestrus cattle. Lower cholesterol level in RB cows was also documented in an earlier report [21]. The serum protein level (g/dl) of normal breeding cows was comparatively higher than those of RB and anestrus cows. However, no statistical significance was observed. This is in agreement with the previous work [18]. Lower serum protein level in RB and anestrus group may lead to deficiency of certain amino acids which are essential for gonadotropin synthesis [22].

## Conclusion

This study revealed a high prevalence of reproductive health problems in the study area. Anestrus, RB, retention of fetal membrane, and dystocia were the major reproductive diseases in crossbred dairy cattle reared under resource poor, smallholders dairy production system in northeastern part of India. Breed, parity and age group are possible risk factors identified for the occurrence of reproductive health problems. Management and fertility of dairy cows among smallholder farms is faced with both challenges and opportunities to improve productivity that are related to feeding, housing, health, and breeding system. Both the challenges and opportunities are influenced by the extent to which farmers have accesses to important services such as extension, health, breeding, and finance. Improvement in service delivery and further capacity building of both farmers and extension staff is required in order to improve dairy management skill and subsequent productivity. Further, detailed research in specific aspects of feeding, housing, health care, and breeding systems would help to identify specific interventions that could be used to improve dairy cattle productivity.

## Authors’ Contributions

MHK: Involved in designing of research, data collection, statistical analysis, and drafted and revised the manuscript. KM: Involved in the collection of data and statistical analysis. SP: Involved in data collection. All authors read and approved the final manuscript.
